# RNAi of the electron transport chain *ATPsynβL* in glutamate neurons protects against age-related intestinal barrier dysfunction.

**DOI:** 10.17912/micropub.biology.002231

**Published:** 2026-06-29

**Authors:** Maria Longenecker, Zoe Clymer, Abigail Forrest, B. Jill Venton, Jeffrey M. Copeland

**Affiliations:** 1 Department of Biology, Eastern Mennonite University, Harrisonburg, VA, US; 2 Department of Chemistry, University of Virginia, Charlottesville, VA, US

## Abstract

Maintenance of the intestinal barrier and abundance of intestinal microbiota are important markers for physiological aging. Glutamate neuron-specific RNAi of the electron transport chain *ATPsynβL* gene has been demonstrated to extend life span and affect daytime sleep behaviors in *Drosophila* . We investigate the intestinal barrier in the *ATPsynβL* RNAi flies and notice a more intact intestinal barrier and fewer intestinal bacteria in late-stage adulthood. These results provide one possible explanation for the prolonged life span in the *ATPsynβL* RNAi flies.

**Figure 1. RNAi targeting ATPsynβL in glutamate neurons delays loss of intestinal permeability in aging Drosophila:** The GAL4 control strain is heterozygous *D42* -GAL4 (B, D) or *Act5C* -GAL4 (C). The *ATPsynβL* control is heterozygous for UAS- *ATPsynβL* -RNAi, while the *ATPsynβL* RNAi line carries both GAL4 and UAS- *ATPsynβL-* RNAi elements. (A) Intestinal integrity was monitored by feeding flies food supplemented with blue food dye. A representative non-Smurf (top) and Smurf (bottom) fly are shown. (B) Intestinal integrity was measured at 5, 30, and 50 days of age, and glutamate neuron-specific RNAi of *ATPsynβL* showed 56 – 62% fewer Smurf flies at 50 days of age. (C) qRT-PCR for flies with ubiquitous knockdown of *ATPsynβL* . (D) Bacterial loads in dissected intestines measured by qRT-PCR of the 16S rRNA gene at 50 days of age. P values of <.05 (*), <.01 (**), <.001 (***), <0.0001 (****), and not significant (ns) are indicated. Error bars show S.D. ranges.

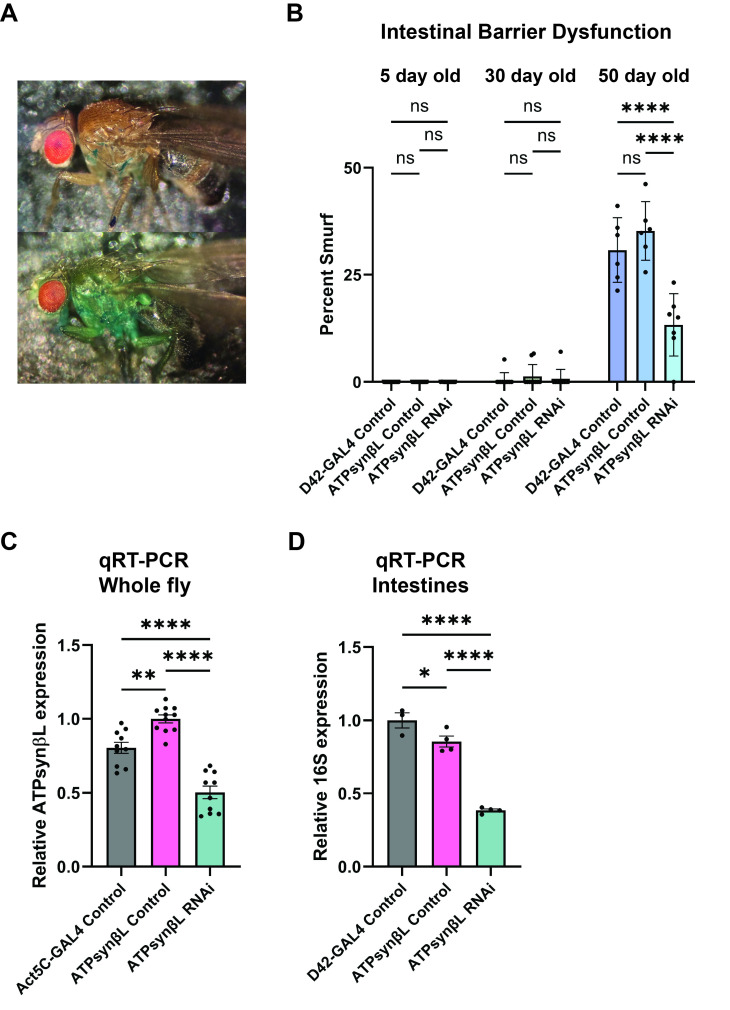



## Description

Aging is generally defined as a progressive loss of physiological function associated with cellular hallmarks, such as telomere attrition, loss of proteostasis, chronic inflammation, and disabled macroautophagy (Lopez-Otin et al., 2023). One particular age-related change observed in zebrafish, mice, and *Drosophila* known to presage death is an increase in intestinal barrier permeability (Zane et al., 2024). This dysfunction of the intestinal barrier co-occurs with changes in the intestinal microbiota, misdifferentiation of intestinal stem cells, and increased levels of inflammation (Salazar et al., 2023). Protective measures aimed at preserving the intestinal barrier have been demonstrated to extend *Drosophila* life span (Salazar et al., 2018; Qin et al., 2025). The Smurf assay is an effective and simple tool used to monitor the integrity of the intestinal barrier, wherein flies are fed food containing 2.5% blue food coloring for 24 hours. Flies with a more permeable intestinal barrier and a higher-risk of mortality, the dye leaks through and turns the entire fly blue (Rera et al., 2011).

The functionality of the mitochondria is well established as important in determining longevity, and tissue-specific knockdown of electron transport chain components has been demonstrated to extend life span in *Caenorhabditis elegans* and *Drosophila melanogaster* (Copeland et al., 2009; Durieux et al., 2011). The role of the electron transport chain in neurons is noteworthy, as robust life span extension has been observed in *Drosophila* when the complex V component *ATPsynβ* L was knocked down in glutamate neurons but not other neuronal subtypes (Keppley et al., 2018; Landis et al., 2023). Glutamate neuron-specific RNAi of *ATPsynβL* was also associated with a pronounced increase in daytime sleep early and midway through adult life (Forrest et al., 2026).

Given the impact that glutamate neuron-specific RNAi of *ATPsynβL* has, we wanted to determine if it also affected the age-related degeneration of the intestinal barrier. We fed the flies blue-dyed food at 5, 30, and 50 days of age. The controls in our experiments included flies heterozygous for the glutamate neuron-specific GAL4 driver ( *D42* ) or the UAS- *ATPsynβL* -RNAi construct. In our investigations, we noticed that loss of the intestinal barrier only occurred later, at 50 days of age, and that glutamate neuron-specific *ATPsynβL* RNAi protected against this barrier loss. Specifically, when we conducted the Smurf assay at 5 and 30 days of age, controls and activated RNAi flies showed a statistically equivalent range of 0 – 1.3% Smurf phenotypes ( Figure 1A, B). At 50 days of age, however, glutamate neuron-specific RNAi of *ATPsynβ* L protected against intestinal barrier dysfunction by 56.5% compared to the control flies ( Figure 1B ).

We validated the functionality of the RNAi construct by measuring *ATPsynβL* expression levels by qRT-PCR. Even though we used the *D42* -GAL4 driver line to test for intestinal barrier dysfunction, we used a different GAL4 line, the ubiquitous *Act5C* -GAL4 driver, to simply test for *ATPsynβL* knockdown. Certainly, these two GAL4 lines do not have the same pattern or strength of gene expression, but the *Act5C* -GAL4 line offers one means of testing RNAi effectiveness on *ATPsynβL* specifically. Using whole flies or excised heads from a *D42* -GAL4 genetic background for our qRT-PCR experiments would contain non-RNAi active cells and provide an inaccurate measurement of *ATPsynβL* knockdown. Immunostaining would require testing with an unproven anti- *ATPsynβL* antibody, a procedure beyond the limited scope of these experiments. In our qRT-PCR experiments, we noticed a significant 38 and 50% knockdown of *ATPsynβL* mRNA transcript from the GAL4 and RNAi control lines, respectively (P < 0.0001) ( Figure 1C ). These results demonstrate that GAL4 activation of the UAS- *ATPsynβL* -RNAi line does specifically knockdown *ATPsynβL* expression.

Changes to the intestinal microbiota are associated with age-related intestinal barrier dysfunction, and an increased number of intestinal bacteria correlates with a shorter life span (Ludington et al., 2025). To measure the changes in bacterial amounts in the *ATPsynβL* RNAi flies, we utilized qRT-PCR with universal primers to the bacterial 16S rRNA gene (Claesson et al., 2010). We limited our qRT-PCR experiments with dissected intestines from 50 day old flies, as the glutamate neuron-specific RNAi did not have any effects on earlier time points in adulthood. Flies with glutamate neuron-specific RNAi of *ATPsynβL* had a 55.1 – 61.6% (P < 0.0001) decrease in bacterial amount, when compared to the control flies ( Figure 1D ).

In this report, we explore the possible connection between the intestinal barrier and longevity in flies with glutamate neuron-specific RNAi of the complex V gene *ATPsynβL* . We observed that activated RNAi led to lower levels of intestinal barrier dysfunction late in the fly life, a change correlated with decreased amounts of intestinal bacteria. It is not surprising that the *ATPsynβL* flies have a more intact intestinal barrier, given the number of reports demonstrating the importance of the intestinal barrier on longevity. Overexpression of occluding junction proteins or induced mitochondrial activity in the intestines have been shown to protect against intestinal barrier dysfunction and dysbiosis as well as extend life span (Salazar et al., 2018; Rera et al., 2011). It is worth noting that we target glutamate neurons to perturb a complex V gene and not the intestines directly. While glutamate neurons are known to innervate the intestinal proventriculus and hindgut, it is a little surprising that they would have such a robust cell non-autonomous effect on intestinal barrier function (Kuraishi et al., 2015). It remains to be seen whether the life span extension we observe in the *ATPsynβL* RNAi flies is mediated solely through the protection of the intestinal barrier or through some other unknown mechanism.

## Methods

**General husbandry**

Flies were fed standard molasses food and reared at 25°C with a 12-hour light:dark cycle. The RNAi line and GAL4 lines were backcrossed a minimum of five times to the *white ^1118^* laboratory strain to minimize hybrid vigor in our studies (Dietzl et al., 2007). The GAL4 control strains were the products of crosses between the *D42* -GAL4 or *Act5C* -GAL4 and our *white ^1118^* strain. The RNAi control strain was the product of a cross between UAS- *ATPsynβL* -RNAi and *white ^1118^* . The activated RNAi line was the result of a cross between the *D42* -GAL4 or *Act5C* -GAL4 and the UAS- *ATPsynβL* -RNAi line.

**Smurf analysis**

To test the integrity of the intestinal tract, a Smurf assay was conducted as previously described (Rera et al., 2012). Twenty females were housed with five males per vial under standard culture conditions and aged to 5, 30, or 50 days of adulthood. Aged flies were fed food containing 2.5% FD&C #1 blue food coloring (Spectrum Chemical, Gardena, CA) for 24 hours and scored for the Smurf phenotype. Measurements were conducted with at least six replicates and at least 230 females.

**Quantitative real-time PCR**

RNA from 5 day old flies was extracted using the RNeasy Mini protocol (Qiagen, Hilden, Germany), and isolated RNA was quantified using a NanoDrop spectrophotometer (Thermo Scientific, Wilmington, DE, USA). 700 micrograms of RNA were reverse transcribed using the iScript cDNA Synthesis kit (Bio-Rad, Hercules, CA). qRT-PCR was performed on a CFX Connect Detection System (Bio-Rad, Hercules, CA) using Sso Advanced Universal SYBR Green Mix (Bio-Rad, Hercules, CA).

Each sample was analyzed with six reactions, along with controls (NTCs) without cDNA in the PCR. The specificity of each amplified reaction was verified by a dissociation curve analysis after each measurement. To determine primer efficiency, serial 2-fold dilutions of each primer set were used to generate a standard curve, and efficiencies (E) were determined based on the slope (M) of the log−linear portion of the standard curve (E = 10−1/M − 1 × 100).

**Statistical analysis**

Data were analyzed using GraphPad Prism (Version 10.5.0 (774), San Diego, CA) and Excel (Microsoft) software. One-way ANOVA tests (Tukey HSD) were run to determine statistical significance.

## Reagents

**Stocks**

The *D42* -GAL4 (RRID: BDSC_8816) and *Act5C* -GAL4 (RRID: BDSC_4414) fly strains were obtained from the Bloomington Stock Center (NIH P40OD018537, Bloomington, IN). The UAS- *ATPsynβL* -RNAi line (VDRC ID: 22112) targeting Complex V of the electron transport chain was purchased from the Vienna Drosophila RNAi Center (Vienna, Austria).

**Primers**

**Table:** 

Primer name	Targeted gene	DNA sequence
JC35	*Act5C* (FBgn0000042)	TTGTCTGGGCAAGAGGATCAG
JC36	*Act5C* (FBgn0000042)	ACCACTCGCACTTGCACTTTC
JC203	*ATPsynβL* (FBgn0036568)	AGGATGAAGCCGAGGATGAG
JC204	*ATPsynβL* (FBgn0036568)	GGAATACCTCCAGCACTAGGTTAGCA
V1F	16S ribosomal DNA	AGAGTTTGATCCTGGCTCAG
V2R	16S ribosomal DNA	CTGCTGCCTYCCGTA

The sequences for the *16S* ribosomal, *ATPsynβL* , and the *Act5C* DNA primers have been previously reported (Forrest et al., 2026; Claesson et al., 2010; Rana et al., 2017).
